# Overcoming legacy system hurdles: a case study on enabling master facility registry interoperability within Ethiopia’s national eHealth architecture

**DOI:** 10.1093/oodh/oqaf031

**Published:** 2025-11-17

**Authors:** Alfa Habib Bushira, Biru Amanuel, Dibaba Legesse, Denboba Wubshet, Kelbessa Asfaw, Kersie Tewodros, Melkamu Gemechis

**Affiliations:** Digital Health, JSI Research & Training Institute, Inc., Sunshine Tower #4, Airport Road, P.O. Box 13898, 1000 Addis Ababa, Ethiopia; Digital Health, JSI Research & Training Institute, Inc., Sunshine Tower #4, Airport Road, P.O. Box 13898, 1000 Addis Ababa, Ethiopia; Digital Health, JSI Research & Training Institute, Inc., Sunshine Tower #4, Airport Road, P.O. Box 13898, 1000 Addis Ababa, Ethiopia; Digital Health, JSI Research & Training Institute, Inc., Sunshine Tower #4, Airport Road, P.O. Box 13898, 1000 Addis Ababa, Ethiopia; Digital Health LEO, Ministry of Health, 1234 Sudan Street, P.O. Box 1234, 1000 Addis Ababa, Ethiopia; Digital Health LEO, Ministry of Health, 1234 Sudan Street, P.O. Box 1234, 1000 Addis Ababa, Ethiopia; Digital Health LEO, Ministry of Health, 1234 Sudan Street, P.O. Box 1234, 1000 Addis Ababa, Ethiopia

**Keywords:** master facility registry, interoperability, legacy systems, health information systems, Ethiopia, eHealth architecture, DHIS2, LMIC

## Abstract

**Introduction:**

Legacy health information systems pose significant barriers to achieving interoperability, which is crucial for strengthening health systems, especially in low- and middle-income countries. Ethiopia, in its pursuit of an ‘Information Revolution’, faced such challenges while working to establish a robust, interoperable Master Facility Registry (MFR).

**Method:**

This case study details a multifaceted approach to overcome these legacy system challenges and integrate the MFR within Ethiopia’s national eHealth Architecture. The methods involved strategically designing and developing the MFR, coupled with implementing a dedicated interoperability layer to facilitate data exchange, primarily with the District Health Information Software 2. Key stakeholders were engaged throughout the process, guided by national eHealth governance structures.

**Result:**

The results demonstrate the successful deployment of a national MFR and achieved interoperability, leading to improvements in health facility data management and accessibility. Challenges related to disparate data sources, varying standards and the need for system-wide coordination were systematically addressed.

**Discussion:**

The successful establishment and integration of a national MFR in Ethiopia, particularly its interoperability with District Health Information Software 2, represents a significant advancement in the country’s health information system capabilities. This achievement is noteworthy given the prevalent legacy system hurdles. The findings indicate that strategic vision, targeted technical solutions and sustained stakeholder engagement were pivotal. The MFR now provides a unified, authoritative source of facility information and its seamless integration with District Health Information Software 2 ensures that routine health data is accurately contextualized geographically and organizationally, addressing data inconsistency and limited accessibility.

**Conclusion:**

The Ethiopian experience offers valuable lessons regarding the strategic importance of a national eHealth vision, targeted technical solutions for interoperability and the necessity of sustained stakeholder collaboration. This initiative underscores the foundational role of an MFR in enhancing HIS performance and provides actionable insights for other low- and middle-income countries striving to modernize their digital health ecosystems despite prevailing legacy constraints.

## INTRODUCTION

### The global imperative for health information system interoperability and the challenge of legacy systems

The capacity of health systems to deliver effective, efficient and equitable care is increasingly reliant on the robust functionality of their Health Information Systems (HISs). A core tenet of modern HIS is interoperability—the ability of different information systems, devices and applications to access, exchange, integrate and cooperatively use data in a coordinated manner [1]. Without such interoperability, health data remains siloed, hindering comprehensive patient care, efficient health system management, informed decision-making and progress toward universal health coverage (UHC) [2]. However, the path to seamless interoperability is often obstructed by the prevalence of legacy systems. These are older, often outdated systems that were developed without current interoperability standards in mind, creating significant technical and operational barriers to data exchange [3]. The challenge posed by legacy systems extends beyond mere technological obsolescence; it often encompasses deeply embedded workflows, established data cultures and pre-existing resource allocation patterns that can prove more formidable to navigate than the technical integration itself. As highlighted in studies of HIS migration, issues such as data integrity concerns stemming from older systems and user resistance to new, integrated platforms are common hurdles [3]. Addressing these socio-technical dimensions is paramount for any successful interoperability initiative.

### The strategic importance of master facility registries in health system strengthening

A Master Facility Registry (MFR), also referred to as a Health Facility Database (HFDB), serves as a cornerstone for a well-functioning national HIS [4]. An MFR is defined as a single, authoritative, comprehensive and continually updated list of all health facilities within a country, encompassing both public and private sectors. Essential data attributes within an MFR typically include a unique identifier for each facility, its official name, administrative location, geographic coordinates, facility type or level, ownership, operational status and an inventory of services offered [4]. The World Health Organization (WHO) strongly advocates for each country to establish and maintain such a singular, authoritative MFR, recognizing its widespread benefits [5]. These benefits include improved data quality through error detection by a wider user base and enhanced data linkage and exchange due to consistent referencing across various information systems [5]. MFRs are foundational for numerous eHealth applications, enabling accurate health service mapping, equitable resource allocation, effective disease surveillance and precise monitoring of health service delivery [4]. In LMICs, where health system infrastructure can be fragmented and data sources disparate, the role of a robust MFR is particularly critical for coherent health system planning and management [4]. The call for a harmonized, sub-Saharan African (SSA) wide HFDB underscores the regional recognition of this need [4].

### Ethiopia’s health system landscape and the ‘information revolution’

Ethiopia’s health system, characterized by a tiered structure aimed at providing comprehensive healthcare services across the nation, faces challenges common to many LMICs, including resource limitations and the need for improved health outcomes. Recognizing the transformative potential of data, the Ethiopian Ministry of Health (MoH) launched the ‘Information Revolution’ as a key agenda within its Health Sector Transformation Plan. The core goal of this initiative is to significantly improve the quality, availability and use of health information for decision-making at all levels of the health system, thereby enhancing the quality, efficiency and accessibility of primary health and nutrition services [6].

Guiding this digital transformation is Ethiopia’s national eHealth Architecture. This architecture serves as a conceptual blueprint outlining the information systems, data sources, standards and integration pathways that the MoH intends to implement and maintain to achieve its strategic health objectives [6]. A central aim of this architecture is to foster data transparency, enable the creation of longitudinal health records and ensure standardized integration across diverse HIS components. The architecture explicitly identifies shared services, including a national MFR and an overarching Interoperability Service, as critical enablers [6]. This national-level strategic commitment to a unified eHealth Architecture creates a distinctively supportive environment for specific digital health projects like the MFR. Unlike initiatives in contexts lacking such a guiding framework, the Ethiopian MFR project benefits from political endorsement and a clear roadmap, which can significantly influence its design, adoption, sustainability and ultimate success in contributing to the broader health system strengthening goals.

### Problem statement: legacy hurdles to MFR interoperability in Ethiopia

Despite the strategic vision provided by the eHealth Architecture, the Ethiopian health information landscape, like many others, has historically been characterized by a variety of legacy systems and data management practices. These presented substantial hurdles to the development and optimal functioning of a truly national, interoperable MFR. Challenges included the existence of multiple, often incomplete and inconsistent, facility lists held by different departments or programs; a lack of standardized unique identifiers for health facilities; the use of outdated technologies for data storage and management; and variable data quality within these pre-existing lists [3]. Furthermore, integrating these disparate sources and establishing interoperability with key national systems, such as the DHIS2 used for routine health reporting, and the electronic Logistics Management Information System (eLMIS), was a complex undertaking. These legacy hurdles directly impacted the efficiency of health information management, complicated planning and resource allocation and limited the ability to gain a unified, real-time understanding of health service availability and capacity across the country.

### Objective of the manuscript

The primary objective of this manuscript is to describe and analyze the process undertaken in Ethiopia to overcome legacy system challenges in the establishment and operationalization of an interoperable national MFR. This includes detailing the MFR’s design, the interoperability architecture implemented, the strategies employed for integration, the outcomes achieved and the critical lessons learned. A secondary objective is to evaluate the MFR’s role as a foundational component within Ethiopia’s national eHealth architecture and its impact on facilitating data exchange with pivotal systems like DHIS2.

## MATERIALS AND METHODS

### Study design and setting

This study employs a descriptive case study methodology to examine a national health informatics implementation project within the Ethiopian public health system. The case focuses on the development, deployment and integration of the national MFR. The project activities described span from the conceptualization phase within the eHealth Architecture to its ongoing operationalization. Key institutional actors involved include the Ethiopian MoH, which provides leadership and governance, and various technical partners, including JSI Research & Training Institute, Inc. through its Data Use Partnership (DUP) project, which provided technical assistance [6].

### The Ethiopian MFR: design and development

The impetus for the development and enhancement of the Ethiopian MFR stemmed directly from the national ‘Information Revolution’ agenda and the strategic imperatives outlined in the eHealth Architecture [6]. The MFR was envisioned as a core shared service, providing a canonical source of truth for all health facility information nationwide.

The scope of the MFR encompasses all types of health facilities, including public and private hospitals, health centers, clinics and health posts. The minimum data elements collected for each facility align with international best practices and WHO recommendations [4], including: a unique national facility identifier, official facility name, administrative location (region, zone, woreda and kebele), geographic coordinates (latitude and longitude), facility type/level, ownership (e.g. public, private for-profit, NGO), operational status (e.g. functional, temporarily closed, under construction) and a list of key services provided.

The initial population of the MFR involved a meticulous process of consolidating data from numerous pre-existing, often fragmented and inconsistent, legacy facility lists maintained by various MoH departments and regional health bureaus (RHBs). This process included extensive data cleaning, validation and de-duplication. While specific de-duplication algorithms were not detailed in the available documentation, the principles of patient-level de-duplication, such as standardizing address information and employing algorithmic techniques with defined thresholds, are conceptually applicable to facility record matching [7]. Field verification and engagement with regional and district-level health officials were also crucial for ensuring data accuracy. The technology platform for the MFR was selected to ensure scalability, security and ease of integration, adhering to the technical standards outlined in the eHealth Architecture [mHealth Evidence Reporting and Assessment (mERA) item 2] [7].

### Interoperability architecture and implementation strategy

A core tenet of the Ethiopian eHealth Architecture is the establishment of seamless data exchange among disparate HIS components. For the MFR, this necessitated the design of an interoperability architecture to facilitate connectivity with key national systems, primarily the District Health Information System version 2 (DHIS2), with future integration planned for systems like the eLMIS and the Human Resources for HIS (HRIS) [6]. The national eHealth Architecture explicitly features an ‘Interoperability Service’ layer, intended to manage critical functions such as authentication, encryption, data routing, transformation, message queuing, validation and translation between connected systems [6].

The technical approach to realize this interoperability involved the development and deployment of a middleware solution, aligning with the ‘Interoperability Service’ concept. This layer leveraged Application Programming Interfaces (APIs) to enable controlled access to MFR data and streamline data exchange with other systems [8]. For the MFR-DHIS2 interoperability, the MFR is embedded with a HAPI-FHIR FHIR server in a hybrid design. The MFR’s .NET-based system pushes facility information to this FHIR server, and the Open Health Information Mediator (OpenHIM)-based interoperability mediator pulls data from the HAPI-FHIR server. This approach aligns with the national eHealth Architecture’s advocacy for the adoption of international standards where practical, recognizing the potential of standards like FHIR in low- and middle-income country (LMIC) contexts [1]. The architecture was designed to accommodate such standards, with data mapping and transformation processes established within the interoperability layer to reconcile variations in data models and coding schemes between the MFR and target systems like DHIS2.

**Figure 1:**
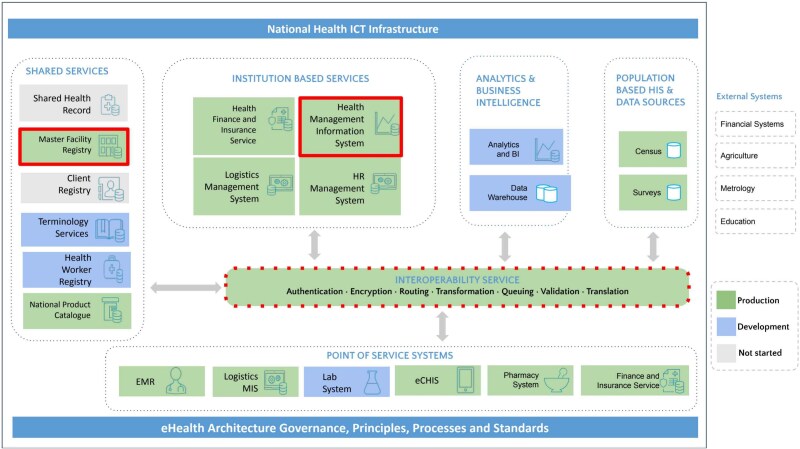
Alignment of MFR-DHIS2 interoperability within Ethiopia’s national eHealth architecture

Figure 1 below illustrates Ethiopia’s National eHealth Architecture, with key systems and components involved in facility data exchange for MFR-DHIS2 interoperability highlighted in red. This visual representation demonstrates how the MFR and DHIS2 align within the broader national architecture, emphasizing the critical pathways and elements that facilitate the seamless flow of facility information as part of the MoH’s strategic vision for digital health.

**Figure 2:**
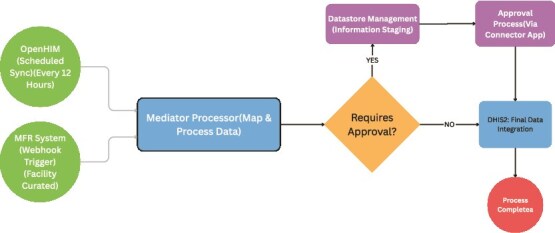
Health facility data Flow from MFR to DHIS2

The implementation strategy unfolded in four key stages, each strategically designed to address specific interoperability challenges while prioritizing data accuracy, scalability and alignment with national HIS priorities:

1. **Facility Data Mapping and Verification**: The initial phase focused on achieving a high degree of concordance between facility records in the MFR and DHIS2 through a dual approach:
  Automated Fuzzy Matching: Algorithms employing fuzzy logic were utilized to identify potential matches between facility records from both systems. This automated process accounted for common data inconsistencies, including spelling variations, differing naming conventions, location mismatches and outdated metadata fields. The primary output was a list of probable matches for subsequent review.
  Manual Mapping and Validation: A dedicated team of digital health and MFR experts conducted a meticulous manual review of unmatched or ambiguously matched facility records. This involved verifying facility authenticity and operational status, correcting inaccurate or incomplete metadata and confirming precise linkages between corresponding records in the MFR and DHIS2. This stage involved an iterative process, initially attempting to map all MFR facilities (over 49 000) to DHIS2 (over 38 000), but refining the strategy to prioritize mapping DHIS2 facilities to the MFR to ensure DHIS2 data quality. Each DHIS2 facility was categorized as Mapped, Partially Mapped, or Not Found, with a collaborative decision-making process involving regulatory teams and DHIS2 experts to register or mark for closure ‘Not Found’ facilities. This comprehensive process yielded an 84% alignment rate, revealing critical discrepancies such as closed facilities still reporting in DHIS2, ‘ghost’ facilities, and inconsistencies in naming or classification.
2. **Development of the OpenHIM-Based Interoperability Mediator**: To enable seamless system-to-system communication, an interoperability mediator was developed based on the OpenHIM platform. This middleware component was designed to actively listen for MFR updates, transform incoming facility data into a DHIS2-compatible standardized structure, and securely queue and route requests for updating facility information within DHIS2. The mediator supported both push (MFR-initiated) and pull (OpenHIM-initiated) mechanisms and incorporated robust error handling, status tracking and detailed logging to ensure data integrity and system stability. Upon receiving facility data, the mediator implemented workflows for new facility registration (pushing structured data to DHIS2) and existing facility updates, with automatic updates for name, last updated date and geographical coordinates and updates requiring approval (via the DHIS2 Connector App) for other attributes as shown in the diagram in Figure 2.
3. **Development of the DHIS2 Connector App**: To finalize the interoperability workflow and enforce data governance within DHIS2, a custom DHIS2 Connector App was developed and deployed. This application provided key functionalities: receiving and registering new/updated facility records from OpenHIM, automatically assigning relevant datasets based on predefined rules (facility type, ownership, settlement and PHCU status), and generating necessary user accounts. Developed using the DHIS2 Web API, the Connector App featured a user-friendly interface for administrators to manage mappings between facility attributes, datasets and user roles. Critically, it served as an approval layer for facility updates staged by the mediator. Designated regional administrators could review and approve these changes through the connector interface, ensuring data accuracy and validity before permanent reflection in DHIS2.
4. **Deployment and Rollout**: The developed interoperability solution underwent rigorous testing in a dedicated sandbox environment using representative facility data. Following successful validation, the system was transitioned to the production environment, hosted on Ministry-managed server infrastructure with comprehensive backup and logging strategies. To ensure long-term sustainability, targeted training was provided to relevant ministry staff and comprehensive documentation was developed. Continuous monitoring mechanisms were implemented to proactively address synchronization issues, data mismatches, or system errors. The production rollout was strategically executed in three phases:
  Phase 1: Initial Migration: Bulk synchronization of all ‘Mapped’ facilities with valid DHIS2 identifiers from the MFR to DHIS2, leveraging the existing reporting hierarchy via JSON payloads and OpenHIM. Bulk assignment of datasets and creation of user accounts followed.
  Phase 2: Daily Synchronization: Configuration of the OpenHIM mediator for daily automated fetching of the reporting hierarchy from the MFR, with automatic synchronization of new or modified facilities to DHIS2 and bulk updates of associated datasets and user accounts. This phase allowed for regulatory review and corrections based on DHIS2 user feedback.
  Phase 3: Standard Workflow Launch: Transition to the steady-state operational workflow with comprehensive training for DHIS2 and regulatory teams on collaborative processes for managing facility data changes. Under this workflow, critical attribute changes were automatically synchronized, while other modifications required manual review and approval via the DHIS2 Connector App by designated administrators, ensuring robust data governance.

### Stakeholder engagement and governance

The MFR and interoperability project operate under the overarching governance framework of the eHealth Architecture and the Information Revolution initiative [6]. This ensures alignment with national health priorities and facilitates coordination among various actors.

A systematic process of stakeholder engagement was crucial throughout the project lifecycle. Key stakeholders included various departments within the MoH, RHBs, Zonal Health Departments, Woreda Health Offices, health facility managers, software developers and implementing partners [9]. Regular consultations, workshops and feedback sessions were conducted to ensure that the MFR design and interoperability workflows met user requirements and addressed concerns. This collaborative approach was vital for securing buy-in, promoting ownership and addressing potential resistance to new systems and procedures, recognizing that early and continuous stakeholder engagement is a strong predictor of project success [8].

Comprehensive training and capacity-building activities were conducted for system administrators responsible for maintaining the MFR and the interoperability layer, as well as for end-users at national, regional and facility levels who would be utilizing the MFR data or interacting with integrated systems (mERA item 10) [10]. This included training on data entry, data quality assurance, system utilization for reporting and planning and basic troubleshooting.

### Ethical considerations and reporting standards

While the MFR primarily deals with facility-level data rather than individual patient data, robust data security, privacy and confidentiality measures were integral to its design and operation (mERA item 14) [10]. This includes secure data storage, controlled access mechanisms, audit trails and protocols for data sharing, aligning with national data protection guidelines and international best practices [11]. Ethical approval from an Institutional Review Board (IRB) was deemed not required for this specific report, as it describes the implementation of a national HIS infrastructure and does not involve direct human subject research or identifiable patient-level data analysis. The focus is on the system’s design, implementation and operational aspects for health system management [11].

This case study has been prepared with consideration for the SQUIRE 2.0 (Standards for QUality Improvement Reporting Excellence) guidelines [12] and relevant items from the mERA checklist [10], adapting them as applicable to a health informatics infrastructure project. No Artificial Intelligence (AI) tools were used in the generation of the core data or the primary analysis presented in this manuscript; AI was utilized solely for assistance in literature review and manuscript formatting, and this usage is hereby disclosed [11].

## RESULTS

### Establishment and features of the national MFR

The implementation efforts culminated in the successful deployment of a national MFR for Ethiopia. This registry now serves as the central, authoritative source for health facility information across the country. As of the latest available data, the MFR includes records for 49 663 health facilities, encompassing public and private sector establishments. A high degree of completeness has been achieved for core data elements, including unique facility identifiers, official names, ownership details, facility type, administrative location and operational status. Significant progress has also been made in capturing and validating geographic coordinates for a vast majority of these facilities, greatly enhancing spatial analysis capabilities.

Key functionalities of the MFR that address previous limitations of fragmented legacy lists include:

- **Standardized Coding and Unique Identifiers:** Each facility is assigned a unique national ID, eliminating ambiguities and facilitating data linkage across different information systems.
- **Web Accessibility:** The MFR is accessible via a web-based platform, allowing authorized users at different levels of the health system to query, view and (with appropriate permissions) update facility information.
- **Defined Update Mechanisms:** Clear protocols and workflows have been established for adding new facilities, updating existing facility information and deactivating closed facilities, ensuring the MFR remains current.
- **Enhanced Data Quality:** Built-in validation rules and regular data quality assessments contribute to maintaining the accuracy and reliability of the facility data.

### Achieved interoperability with DHIS2 and other systems

A primary achievement of this initiative has been the establishment of robust interoperability between the MFR and Ethiopia’s national DHIS2 instance. This integration allows for the automated synchronization of the official health facility list from the MFR into DHIS2, ensuring that the organizational unit hierarchy within DHIS2 accurately reflects the recognized health facilities in the country [13]. This is critical for accurate health service data reporting, analysis and resource allocation based on DHIS2 data.

The types of data exchanged primarily include the core facility master data (unique IDs, names, locations, types, ownership, settlement and reporting hierarchy) from the MFR to DHIS2. This ensures that all health program data reported through DHIS2 can be correctly attributed to an officially recognized and uniquely identified health facility. While direct interoperability with other systems like eLMIS and HRIS is part of the broader eHealth Architecture vision [6], the initial focus has been on solidifying the MFR-DHIS2 link. Metrics related to the data exchange, such as the frequency of synchronization (e.g. daily, weekly) and the success rate of data updates, indicate a reliable and consistent flow of information. For example,.

### Overcoming legacy system hurdles

The project successfully addressed several critical legacy system challenges:

- **Unification of Disparate Facility Lists:** Multiple, often conflicting, facility lists from various departments and older databases were consolidated into the single MFR, creating one source of truth.
- **Resolution of Data Inconsistencies:** Through intensive data cleaning, validation and stakeholder consultation, inconsistencies in facility names, locations and classifications present in legacy data were largely resolved.
- **Integration of Isolated Data Silos:** The MFR, through the interoperability layer, now serves as a central hub for facility information, breaking down previous data silos and enabling consistent facility identification across systems. For example, where previously program-specific facility lists might have used different naming conventions or lacked unique IDs, the MFR now provides a standardized reference. The challenge of integrating data from proprietary systems with open-source platforms like DHIS2 was navigated by developing specific connectors and transformation routines within the middleware [6], rather than attempting direct point-to-point integrations with each legacy source.

### Impact on data management and system efficiency

While a formal, comprehensive impact evaluation is ongoing, initial observations and user feedback indicate notable improvements:

- **Improved Data Quality:** The accuracy, completeness and timeliness of the national facility list have demonstrably improved. For instance, there has been a notable improvement in the percentage of facilities with validated Global Positioning System (GPS) coordinates, reaching 87%.
- **Enhanced Efficiency:** Health system managers and planners at national and regional levels report significantly reduced time and effort required to compile accurate facility lists for planning, budgeting and emergency response. Access to an up-to-date, web-based MFR has streamlined these processes, leading to preliminary impacts on health system performance and planning. For instance, the improved availability of reliable and consistent facility data supports more precise resource allocation, such as the deployment of vaccines or medical supplies, and facilitates better targeting of health interventions. This enhanced data foundation indirectly contributes to improved patient care by ensuring that services and resources are distributed more equitably and effectively across the country.
- **User Feedback:** Informal feedback collected during training sessions and user support interactions suggests a positive reception of the MFR and its integration with DHIS2, with users appreciating the improved data consistency and ease of access (mERA item 7).

### Challenges encountered during implementation and mitigation

The journey was not without its obstacles. Several challenges, common in large-scale health informatics projects in LMICs, were encountered:

- **Technical Challenges:** Ensuring reliable data exchange between systems with different underlying technologies and data standards required significant technical effort. Initial issues with data transformation logic and API version compatibility needed iterative refinement. The ‘middleware solution’ approach, while effective, required specialized skills for development and maintenance [6].
- **Operational Challenges:** Maintaining data quality requires continuous effort, including regular updates from the field and periodic data audits. Ensuring that all relevant stakeholders consistently use the MFR as the primary source and adhere to update protocols remains an ongoing operational focus.
- **Data Governance and Standards:** While the eHealth Architecture provides a framework, the practical enforcement of data standards across all legacy and new systems and ensuring adherence to data governance policies, required persistent advocacy and coordination. The issue of ‘different health data standards between the two systems’ (MFR and potentially other source systems like LIS) was a notable challenge [6].
- **Human Resource Capacity:** Building and retaining skilled personnel for MFR administration, interoperability layer management and data analysis is a continuous need.

Mitigation strategies included phased implementation, iterative development with frequent stakeholder feedback loops, dedicated technical support teams, comprehensive training programs and strong leadership and coordination from the MoH. Transparently reporting these challenges and the adaptive strategies employed is crucial, as it offers a realistic portrayal of the implementation journey and provides valuable, practical lessons for other countries. This approach moves beyond a simplistic success narrative to a more nuanced understanding of the complexities involved in such endeavors as Table 1 shows in detail the comparison of facility data management before and after MFR interoperability.

**Table 1:** Comparison of facility data management metrics before and after MFR interoperability implementation

**Metric**	**Baseline (before MFR/interoperability) (estimate)**	**Post-implementation (1 year after full MFR-DHIS2 interop.) (actual/estimate)**	**Data source(s)**
Completeness of national facility master list (%)	Approx. 70%	>95%	MoH records, MFR database
Accuracy of facility location data (GPS validated) (%)	<50%	>90%	MFR database, field verification reports
Time to generate national facility report for planning (person-days)	10–15 person-days	<1 person-day	User reports, MoH planning department feedback
Number of discrepant facility records across key systems (e.g. MFR vs. old program lists)	High (unquantified, est. >1000s)	Low (regularly reconciled, <50 discrepancies at any point)	MFR audit logs, DHIS2 org unit validation reports
DHIS2 org unit hierarchy alignment with MFR (%)	Approx. 60% (manual, inconsistent updates)	> 99% (automated synchronization)	DHIS2 admin logs, MFR synchronization logs

## DISCUSSION

### Interpretation of key findings

The successful establishment and integration of a national MFR in Ethiopia, particularly its interoperability with DHIS2, represents a significant advancement in the country’s HIS capabilities. This achievement is particularly noteworthy given the prevalent legacy system hurdles, such as fragmented data sources and lack of standardization, that often impede such initiatives in LMIC contexts [3]. The findings indicate that a combination of strategic vision (the eHealth Architecture), targeted technical solutions (the interoperability layer) and sustained stakeholder engagement were pivotal. The MFR now provides a unified, authoritative source of facility information and its seamless integration with DHIS2 ensures that routine health data is accurately contextualized geographically and organizationally. This directly addresses the critical problems of data inconsistency and limited accessibility that previously hampered effective health system management.

### Alignment with national and global digital health strategies

The MFR initiative is deeply embedded within and demonstrably advances the goals of the country’s ‘Information Revolution’ and its national eHealth Architecture [6]. By creating a foundational ‘shared service’, the MFR supports the broader architectural principles of data transparency, standardization and efficient data exchange. Globally, this work aligns closely with WHO recommendations for all countries to maintain a single, geocoded, authoritative MFL [5] and contributes to the global push for greater HIS interoperability to improve health outcomes [1].

Utilizing the WHO Classification of Digital Health Interventions (DHIs) v1.0 [19], the Ethiopian MFR can be classified primarily under **Interventions for health system or resource managers**, specifically as **DHI 3.7 Facility management**, with the detailed intervention being **3.7.1 List health facilities and related information**. The interoperability component, particularly the middleware and API infrastructure enabling data exchange with DHIS2 and other systems, falls under **Interventions for data services**, specifically **DHI 4.4 Data exchange and interoperability**, with the detailed intervention **4.4.1 Data exchange across systems**. Adopting such standardized classifications is important; it not only provides a common lexicon for describing the intervention but also facilitates comparison with, and learning from, similar initiatives globally, thereby enhancing the international relevance and understanding of Ethiopia’s achievements [20].

### Addressing legacy system hurdles: lessons for LMICs

Ethiopia’s experience offers valuable insights for other LMICs grappling with similar legacy system challenges. Key effective strategies included:

1. **A Phased and Prioritized Approach:** Rather than attempting to overhaul all legacy systems simultaneously, the focus on establishing the MFR as a foundational element and then prioritizing its interoperability with a high-impact system like DHIS2 proved effective.
2. **Development of a Centralized Interoperability Layer:** Instead of numerous point-to-point integrations, the creation of a middleware or interoperability service layer provided a more manageable and scalable solution for connecting disparate systems, including those with proprietary elements [6]. This aligns with the concept of service-oriented architectures discussed in the context of eHealth in Ethiopia previously [22].
3. **Strong Stakeholder Buy-in and National Ownership:** Continuous engagement with all levels of the health system, from national policymakers to facility-level users, fostered ownership and helped navigate resistance to change, a common issue with legacy system transitions [3].
4. **Pragmatic Data Standards Adoption:** While international standards like FHIR are aspirational [10], the initial implementation focused on achieving functional interoperability using existing capacities and adaptable solutions, with a pathway toward greater standards adherence over time.

These approaches resonate with broader discussions on overcoming eHealth implementation challenges in LMICs, which often highlight issues of infrastructure, human capacity and the need for contextually appropriate solutions [10].

### The role of governance, policy and partnerships

The success of the MFR initiative cannot be divorced from the enabling environment created by Ethiopia’s national eHealth governance structures and clear policy direction stemming from the Information Revolution [6]. This top-level commitment provided the mandate and framework necessary for such a large-scale undertaking. Furthermore, effective partnerships between the MoH, technical assistance providers like JSI DUP and other development partners were crucial for bringing in specialized expertise, resources and supporting capacity development efforts [6]. Clear policies on data standards, data sharing agreements and system access rights are fundamental underpinnings for sustainable interoperability.

### Implications for health system performance and UHC

An accurate and interoperable MFR has profound implications for health system performance and the journey toward UHC. It enables more precise planning of health service delivery, equitable allocation of resources (human, financial and material) and more effective monitoring of service availability and readiness [2]. For example, by linking MFR data with DHIS2, health managers can better understand population coverage for various interventions, identify underserved areas and track progress toward health targets. This improved information ecosystem is a critical enabler for evidence-based decision-making, which is essential for strengthening primary healthcare and achieving UHC goals. The benefits extend to improved public health surveillance, emergency preparedness (as seen with the COVID-19 response leveraging similar architectural components [6]) and overall health system resilience.

### Sustainability and scalability considerations

Long-term sustainability and scalability of the MFR and its interoperability services are paramount [14]. Key factors in the Ethiopian context include:

- **Institutionalization within MoH:** Ensuring the MFR is fully owned, managed and budgeted for by the MoH is critical.
- **Continuous Capacity Building:** Ongoing training and support for personnel at all levels to manage, use and update the MFR and interconnected systems.
- **Technical Maintenance and Upgrades:** A plan for regular maintenance, software updates and adaptation to new technologies and standards.
- **Robust Data Governance:** Continued enforcement of data quality protocols and update mechanisms.
- **Sustainable Financing:** Allocating sufficient domestic resources for the operational costs of the MFR and the interoperability infrastructure. The mERA checklist item regarding ‘Limitations for delivery at scale’ [10] prompts consideration of factors like internet connectivity in remote areas, availability of skilled IT personnel across all regions and the financial resources required for nationwide upkeep. While significant progress has been made, these remain areas for ongoing attention to ensure the system’s benefits reach every part of the country.

### Limitations of the initiative and the case study

While the Ethiopian MFR initiative represents a significant step forward, certain limitations exist. Data quality, though vastly improved, remains an area requiring continuous vigilance and effort, particularly for certain data elements or in specific geographic areas. Full interoperability with all desired HIS components as envisioned in the eHealth Architecture [e.g. comprehensive integration with eLMIS, HRIS and Civil Registration and Vital Statistics (CRVS)] is a longer-term goal that is being pursued incrementally.

This case study, by its nature, provides an in-depth look at one national experience. While many lessons are transferable, the specific contextual factors of Ethiopia (e.g. its particular governance structure, the Information Revolution agenda) mean that direct replication in other settings may require adaptation. The study primarily relies on project documentation, architectural diagrams and qualitative reports; a more extensive quantitative impact assessment on health outcomes or system efficiency metrics would require further dedicated research. Acknowledging these limitations is important for scientific rigor and provides a balanced perspective on the achievements and the path still ahead [12]. Table 2 describes in detail the lessons learned from the MFR interoperability and recommendations.

**Table 2:** Lessons learned from the MFR interoperability project and recommendations for LMICs

**Domain**	**Key lesson learned from Ethiopia**	**Specific recommendation for other LMICs**
Technical	A dedicated interoperability layer/middleware is more scalable and manageable than multiple point-to-point integrations.	Invest in or develop a central interoperability platform/service bus to mediate data exchange, rather than relying solely on direct system-to-system connections, especially when dealing with a mix of legacy and new systems.
	Standardizing facility identification (unique IDs) is a non-negotiable prerequisite for MFR functionality and interoperability.	Prioritize the establishment of a national unique facility identification system early in the MFR development process. Ensure this ID is adopted and used across all relevant HISs.
Governance & policy	A national eHealth strategy and architecture provide essential guidance, mandate and coordination for complex projects.	Develop and endorse a comprehensive national eHealth/digital health strategy and architecture that clearly defines the role of foundational systems like MFRs and outlines interoperability principles and standards. Ensure strong government leadership and ownership.
	Clear data governance policies are crucial for managing shared data resources like an MFR.	Establish clear policies for MFR data access, sharing, updates, quality assurance and security. Define roles and responsibilities for MFR management and maintenance at all levels of the health system.
Stakeholder engagement	Continuous and broad stakeholder engagement from national to local levels is vital for buy-in, adoption and sustainability.	Implement a structured and ongoing stakeholder engagement plan, involving policymakers, program managers, IT staff, healthcare providers and private sector/NGO representatives throughout the MFR lifecycle (design, development, deployment, maintenance).
Capacity building	Sustained investment in human resource capacity is necessary for system management, data use and technical support.	Develop a long-term capacity building plan that addresses the skills needed for MFR administration, data quality management, use of MFR data for decision-making, and technical maintenance of the MFR and interoperability components.
Sustainability	Phased implementation focusing on high-impact integrations (like MFR-DHIS2) can demonstrate value and build momentum.	Prioritize interoperability use cases that deliver clear and quick benefits to end-users and decision-makers. Start with a manageable scope and scale up incrementally as capacity and resources allow. Secure domestic budget allocations for long-term operational costs.

## CONCLUSION

### Summary of main findings

This case study has detailed Ethiopia’s journey in establishing a national, interoperable MFR by systematically addressing the complex challenges posed by legacy HISs. Operating within the strategic framework of its national eHealth Architecture and the ‘Information Revolution’ agenda, Ethiopia successfully deployed an MFR that now serves as the authoritative source for facility data. Crucially, robust interoperability was achieved, particularly with the DHIS2 platform, through the implementation of a dedicated interoperability service layer. This has led to significant improvements in the consistency, accessibility and utility of health facility information, which is foundational for effective health system management.

### Significance and contribution

The Ethiopian MFR initiative is a testament to how LMICs can make substantial progress in modernizing their digital health infrastructure despite resource constraints and the complexities of existing legacy environments. The key contribution of this work lies in demonstrating a pragmatic and strategically guided approach to building essential HIS components. The lessons learned regarding technical design, governance, stakeholder engagement and phased implementation hold considerable relevance for other LMICs embarking on similar digital transformation journeys. This experience provides a tangible example of how a foundational system like an MFR can be leveraged to enhance data-driven decision-making and support the broader goals of health system strengthening and achieving UHC.

### Future directions/outlook

The journey of Ethiopia’s MFR and its integration into the broader eHealth ecosystem is ongoing. Future directions will likely include extending interoperability to other critical systems such as eLMIS, HRIS and CRVS systems, as envisioned in the national eHealth Architecture. Continuous efforts will be needed to enhance MFR data quality, expand its use for advanced analytics and geospatial mapping and ensure its long-term sustainability through capacity building and domestic resource allocation. Further research could focus on quantifying the impact of the interoperable MFR on specific health system performance indicators and patient outcomes.

### Concluding ‘take-away’ message

Overcoming legacy system hurdles to achieve HIS interoperability is a complex but achievable endeavor. Ethiopia’s experience with its MFR demonstrates that a clear national vision, coupled with appropriate technical solutions, strong governance and collaborative partnerships, can pave the way for a more integrated and data-driven health system, offering valuable insights for the global digital health community.

## Data Availability

The specific dataset of the Ethiopian Master Facility Registry is managed by the Ministry of Health and is subject to national data sharing policies. Aggregated data and descriptions of the system architecture are based on publicly available reports and project documents as cited. Further inquiries regarding data access may be directed to the Ethiopian Ministry of Health.
